# Effectiveness of Comirnaty^®^ Vaccine and Correlates of Immunogenicity and Adverse Reactions: A Single-Center Prospective Case Series Study

**DOI:** 10.3390/vaccines10081170

**Published:** 2022-07-22

**Authors:** Diego Fernández-Lázaro, Manuel Garrosa, Nerea Sánchez-Serrano, Evelina Garrosa, Elena Jiménez-Callejo, María Dolores Pardo Yanguas, Juan Mielgo-Ayuso, Jesús Seco-Calvo

**Affiliations:** 1 Department of Cellular Biology, Histology and Pharmacology, Faculty of Health Sciences, Campus of Soria, University of Valladolid, 42004 Soria, Spain; nsanchezser@saludcastillayleon.es (N.S.-S.); ejimenezca@saludcastillayleon.es (E.J.-C.); neparyan@hotmail.com (M.D.P.Y.); 2 Neurobiology Research Group, Faculty of Medicine, University of Valladolid, 47005 Valladolid, Spain; garrosa@med.uva.es; 3 Department of Cell Biology, Genetics, Histology and Pharmacology, Faculty of Medicine, and Institute of Neurosciences of Castile and Leon (INCYL), University of Valladolid, 47005 Valladolid, Spain; evelinags17@gmail.com; 4 Microbiology Unit of Santa Bárbara Hospital, Castile and Leon Health (SACyL), 42003 Soria, Spain; 5 Preventive Medicine Service of the Santa Bárbara Hospital, Castile and Leon Health (SACyL), 42003 Soria, Spain; 6 Emergency Medicine Service of the Santa Bárbara Hospital, Castile and Leon Health (SACyL), 42003 Soria, Spain; 7 Department of Health Sciences, Faculty of Health Sciences, University of Burgos, 09001 Burgos, Spain; fmielgo@ubu.es; 8 Physiotherapy Department, Institute of Biomedicine (IBIOMED), Campus of Vegazana, University of Leon, 24071 Leon, Spain; dr.seco.jesus@gmail.com; 9 Psychology Department, Faculty of Medicine, Basque Country University, 48900 Leioa, Spain

**Keywords:** elderly, healthcare workers, BNT162b2, SARS-CoV-2, humoral response, adverse effects, immunogenicity, case report

## Abstract

The literature suggests that real-world data on the effectiveness and safety of the BNT162b2 vaccine depend on the characteristics of the vaccinated volunteers. The purpose of this study was to evaluate antibody responses and kinetics, established association with sociodemographic and clinical characteristics, and adverse reactions after complete vaccination with the BNT162b2 vaccine. A single-center prospective case series study was conducted with 112 eligible volunteers who were institutionalized elderly and health care workers with had a negative anti-SARS-CoV-2 IgG test prior to receiving the first dose of vaccine. At least one serological antibody test after each dose of vaccine was performed. Volunteers with a positive SARS-CoV-2 PCR test before vaccination were excluded. A chemiluminescent immunoassay anti-S1 antibody assay performed a serological evaluation. Both vaccine doses elicited positive IgG antibodies 3799.0 ± 2503.0 AU/mL and 8212.0 ± 4731.0 AU/mL after 20 days of the first and second doses of BNT162b2, respectively. Comirnaty^®^ vaccine induced an immune response with antibody production against SARS-CoV-2 in 100% of participants, regardless of age (Spearman rho = −0.10, *p*-value = 0.312), body mass index (Spearman rho = 0.05, *p*-value = 0.640), blood group first dose (*p*-value for Kruskal–Wallis test = 0.093) and second dose (*p*-value for Kruskal–Wallis test = 0. 268), number of drugs (Spearman rho = −0.07, *p*-value = 0.490), and number of chronic diseases first dose (*p*-value for Kruskal–Wallis test = 0.632) and second dose (*p*-value for Kruskal–Wallis test = 0.510). IgG antibodies to SARS-CoV-2 were intensely elevated after the second administration of the BNT162b2 vaccine. The higher the titer of anti-peptide IgG antibodies generated after the first dose of vaccine, the higher the titer generated by the second dose of vaccine (Spearman rho = 0.86, *p*-value < 0.001) and the total antibody titer (Spearman rho = 0.93, *p*-value < 0.001). Furthermore, no serious adverse effects were reported among participants, although mild to moderate adverse effects (local or systemic) were reported after both doses of the BNT162b2 vaccine, being more frequent after the first dose of the vaccine. No participants showed a positive PCR. The BNT162b2 vaccine induces a robust and rapid antibody response regardless of participant characteristics. The second dose might be especially important because of the increased immunogenicity it produces and the possible temporal distancing of the interval between doses. In general, the vaccines were well tolerated.

## 1. Introduction

Since the end of 2019, Severe Acute Respiratory Syndrome-2 (SARS-CoV-2) continues to cause a multisystem illness named coronavirus disease 2019 (COVID-19), which primarily causes respiratory symptoms [1]. SARS-CoV-2, with devastating consequences for the health of mankind, has transformed many things in our daily lives: it has affected the way we live our leisure time, play sports, work, and socialize [2]. At least 500 million cases of COVID-19 and six million deaths because of it have been reported [3]. Previously the main therapies to treat the disease have been antivirals, anti-inflammatory drugs, dexamethasone, and respiratory therapy. Currently, other therapeutic strategies, including convalescent plasma therapy, monoclonal antibodies, immunoglobulin therapy, and cell therapy, have been shown to be effective against the virus. However, there is still no specific and approved option for treating SARS-CoV-2 infection, and further studies are needed to determine the safety and efficacy of current treatment strategies [4].

Different interventions in the form of several layers of protection prevent the spread of SARS-CoV-2. However, no intervention is perfect; each has flaws, and when these align, the risk of contagion increases [5]. Vaccination will add another layer of protection because it is the most effective intervention to deal with the COVID-19 pandemic by establishing herd immunity among the general population [6]. The World Health Organization (WHO) encourages vaccination against COVID-19 to stop the COVID-19 pandemic [7]. According to the R0 for the SARS-CoV-2 Alpha variant (2.5–3.5), it would be necessary to immunize 60–70% of the world population [8]. However, the appearance of more contagious SARS-CoV-2 variants is changing the paradigm for achieving herd immunity. Thus, with the Delta variant (R0 = 7.5–9.5) and Lambda variant (R0 ≈ 10), it would be necessary to immunize more than 80–90% of the world population [9,10]. For the BA.1 omicron variant (R0 ≥ 9.5), BA.2 omicron variant (R0 ≥ 13.3) and BA.4/BA.5 (R0 ≥ 18) [7], the threshold for herd immunity against the omicron variable at 95% of the population [11]. However, these immunization thresholds ≥80% of the population are considered “unattainable” by vaccination [12]. In many countries, vaccination campaigns do not allow such high percentages of the population to be vaccinated. Furthermore, there are people who cannot be vaccinated because they suffer from some type of health problem and others who do not want to be inoculated of their own free will.

To date, five COVID-19 vaccines have been approved in Spain by the European Medicines Agency (EMA) and the Spanish Agency for Medicines and Health Products (AEMPS). The five vaccines were manufactured using the following technologies: (i) mRNA: mRNA-1273 (Moderna/Spikevax^®^), BNT162b2 (Pfizer-BioNTech/Comirnaty^®^); (ii) viral vector: Ad26.COV2.S (Janssen-Johnson & Johnson, Belse, Belgium), AZD1222 (Oxford-AstraZeneca/Vaxzevria^®^); (iii) protein: recombinant spicule or spike (S) protein nanoparticle vaccine combined with the Matrix-M adjuvant (Novavax/Nuvaxovid^®^) [13]. These vaccines cause the immune system to act against the S protein of SARS-CoV-2, generating specific antibodies. Nevertheless, in SARS-CoV-2 variants with mutations in the S protein, vaccine immunity would be compromised [14].

The BNT162b2 vaccine uses single-stranded messenger RNA constructs, capped at the 5′ end, which encodes the complete SARS-CoV-2 viral spike (S) protein, with two amino acid changes that maintain it in the prefusion conformation [15]. The formulation of the mRNA in lipid nanoparticles allows for its entry into host cells without degradation. Expression of the genetic information by the cellular machinery produces the SARS-CoV-2 S protein, which is displayed on the cell surface [16]. Detection of this antigen induces an immune response against the S antigen, including both neutralizing antibodies and cell-mediated immunity, which is the basis of protection against COVID-19. Because this vaccine does not contain the whole live virus, it cannot produce disease. Naturally, the mRNA is degraded within a few days [17]. BTB162b2 vaccine was between 90% and 100% effective in preventing SARS-CoV-2 infection, with minimal adverse events, such as fatigue, drowsiness, pain at the injection site, and mild to moderate headaches [14].

In Spain, a country with a population of about 46 million, 12 million cases of COVID-19 with 105 thousand deaths were reported by 12 April 2022 [3]. On 12 April 2022, 95 million doses of COVID vaccines were administered in Spain, which meant that 39 million people, 92.5% of the population over 12 years of age, received two doses of vaccine, of which 27 million people were administered BTB162b2 vaccine [18]. Spain’s vaccination strategy, in stage one (first available doses), prioritized: institutionalized patients and the staff in nursing homes or long-term care facilities; healthcare workers who worked on the front line and treated COVID-19 patients; and non-institutionalized major dependents [19]. Vaccine efficacy and antibody response to first and second doses of vaccine in susceptible populations and healthcare workers are crucial for the scientific community to know. Especially because of the possibility, raised in some governments, of administering a booster dose of COIVD-19 vaccine in a situation of a vaccine shortage. Therefore, there is a need to study the efficacy of vaccines in real uncontrolled conditions, especially in a clinically vulnerable and healthcare-relevant population. In this study, we reported the antibody response in health care workers and institutionalized patients, the association established between antibody titer and sociodemographic (age, body mass index), and clinical (drug treatments, chronic conditions, blood group) characteristics after vaccination with a full schedule (two doses) of BNT162b2 vaccine. In addition, adverse reactions and the association of the kinetics of antibody-mediated immunity after the first and second doses of the BNT162b2 vaccine were evaluated.

## 2. Material and Methods

### 2.1. Study Design

**Figure 1:**
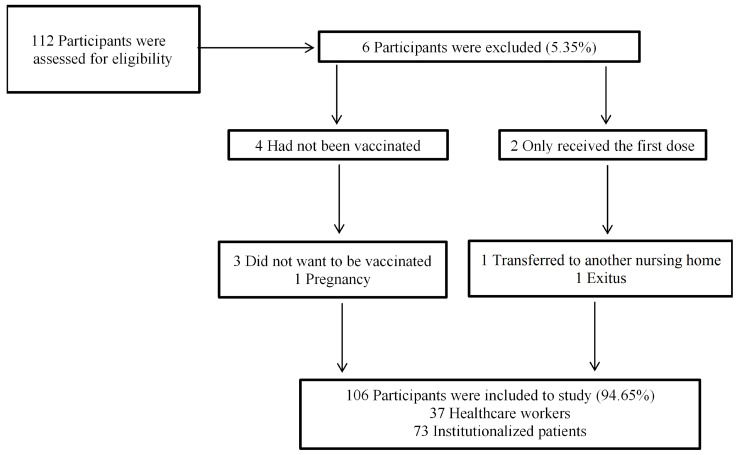
Flowchart for sample selection.

**Figure 2:**
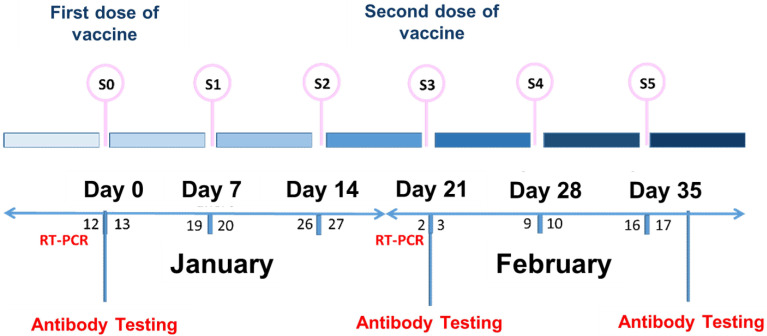
Timeline—Historical and current information from this a single-center prospective case series study.

A total of 112 participants were invited to participate in a longitudinal prospective monocentric observational study performed at the Mixed Nursing Home in Soria (Spain), which evaluates clinical and immunologic responses to the Pfizer BioNTech (BNT162b2) COVID-19 vaccine (Conmirnaty^®^). The cohort consisted of generally healthy adults aged ≥ 18 years who were institutionalized patients and healthcare workers. All study participants also met the following criteria (i) seronegative for SARS-CoV-2 anti-S protein before the first dose of vaccine; (ii) no previous confirmed history of SARS-CoV-2 infection by RT-PCR before each dose of vaccine or COVID-19 infection during the study; (iii) have received the full schedule (two doses) of BNT162b2 vaccine; (iv) have completed the vaccination symptom questionnaires within 21 days of each dose of vaccine received. Out of the total of 112 participants invited to participate, 4 healthcare workers were excluded (3 because they refused to be vaccinated and 1 because of pregnancy), 2 institutionalized patients were excluded because they only received the first dose of the vaccine (exitus and transfer to another nursing home). Therefore, the study sample size consisted of 106 participants (Figure 1). This study was conducted following the CARE guidelines (for CAse REports) (https://www.care-statement.org/ (accessed on 7 June 2022)) [20]. The temporal information of this study is depicted in Figure 2 as a timeline, which allows for historical and current information of this single-center prospective case series study.

### 2.2. Vaccination Protocol

All participants received two doses of the BNT162b2 vaccine. The vaccine used in this study was manufactured by Pfizer/BioNTech. The BNT162b2 vaccine is created by in vitro transcription from a DNA template in a lipid nanoparticle medium with the single-stranded mRNA formulation capped at the 5′ end, which allows it to enter host cells without degradation. Each 0.3 mL dose contains 30 μg of purified mRNA in lipid nanoparticles [21]. In participants with no prior evidence of COVID-19, two doses of the vaccine were administered 21 days apart by intramuscular injection (deltoid) of the non-dominant arm.

### 2.3. Antibody Testing

Detection of IgG against the receptor-binding domain (RBD) region of the S1 subunit of the SARS-CoV-2 spicule (S) protein in serum and plasma was performed using the SARS-CoV-2 IgG II Quant assay (Abbott, Abbott Park, Chicago, IL, USA) [22]. Antibody against the SARS-CoV-2 spike protein determination was performed at three points in the assay, the day before the start of vaccination and 20 days after receiving each of the two doses of BNT162b2 vaccine. Thus, the SARS-CoV-2 IgG II Quant assay allowed for the evaluation of the immunity status of the study participants (vaccinated against COVID-19) by monitoring the antibody response in individuals and quantitatively measuring IgG antibodies against the RBD of the SARS-CoV-2 spicule.

### 2.4. SARS-CoV-2 Virus Diagnosis

The clinical diagnosis of active SARS-CoV-2 infection was performed by RT-PCR following the methodology described by Fernández-Lázaro et al. [23]. RT-PCR testing was performed at 3 time points during the study, on the days before each anti-SARS-CoV-2 IgG antibody test.

### 2.5. Assessment of BNT162b2 Vaccine-Related Adverse Reactions

Participants completed a vaccine-associated symptom questionnaire within 21 days of each vaccine dose received. The questionnaires asked about the presence and severity of a total of 11 potential adverse reactions, 3 considered local reactions (injection site pain, injection site redness, injection site swelling), and 8 considered systemic reactions (chills or shivering, fatigue or tiredness, muscle aches or pains, headache, joint pains, vomiting or nausea, diarrhea, fever ≥ 38.0 °C). The severity of each symptom was defined as the intensity of the symptom and was evaluated on a numerical scale from 1 to 4 (1 = mild, 2 = moderate, 3 = serious, 4 = severe).

### 2.6. Data Collection

Two study investigators (D.F.-L. and C.I.F.-L.) examined electronic medical records and performed specific tests designed for this study. Measures included in the data collection were sociodemographic and lifestyle, physical fitness, and clinical characteristics (Table 1).

#### 2.6.1. Sociodemographic and Lifestyle

Gender, age, nationality (Spanish or other), body mass index (BMI), tobacco consumption (smokers, ex-smokers, or never smokers), adherence to the Mediterranean diet, and self-perception of health status were included as sociodemographic and lifestyle characteristics. BMI was calculated according to Spanish Obesity Society (SEEDO) criteria [24], adherence to the Mediterranean diet was evaluated with a score using the 14-item questionnaire proposed by Trichopoulou et al. [25], and self-perception of health status was assessed employing a self-made visual analog scale adapted from Gould et al. [26].

#### 2.6.2. Physical Fitness

Manual pressure dynamometry was evaluated, in both hands, by performing two measurements with an electronic hand dynamometer (CAMRY MO. EH101, General ASDE, Madrid, Spain), starting with the dominant hand and with the arm in functional position, taking the highest value for each hand [27]. The risk of falling was evaluated through the “*Get Up and Go Test*”, a test proposed by Gálvez Cano et al. [28], in which the time required to get up from the chair, walk to a mark located 3 m away, turn around and sit back in the chair was considered, thus assessing the agility, balance, and resistance of the participants. When the participant’s time exceeded 30 s, a fall risk was considered [28].

#### 2.6.3. Clinical Characteristics

Data about clinical characteristics included as presence of known drug allergies (yes/no); Previously passed COVID-19 infection (yes/no); Pathologies (arterial hypertension, obesity, insulin-dependent diabetes mellitus, respiratory, cancer, and cardiovascular); Usual treatment (antihypertensives, anticoagulants, immunosuppressants, anxiolytics or sedatives, hypolipidemic agents, antidiabetics and cardiovascular); Use of oxygen therapy (current, previous/occasional or never); vital signs (blood pressure [BP], heart rate [HR], temperature [T°] and oxygen saturation [O_2_] at rest were measured with Vital Signs Monitor RVS-100 (RIESTER, Jungingen, Germany), before vaccination and after receiving each of the vaccine doses; Blood group (A, B, AB, O).

#### 2.6.4. Vaccination

Vaccination processes were collected longitudinally at each vaccine dose and included the vaccination center (institution or hospital), vaccine lot, and vaccination site (dominant arm and non-dominant arm).

### 2.7. Data Management and Statistical Analysis

The information collected on the study participants was coded using Excel spreadsheets and exported to the IBM-SPSS statistical program (version 23.0) for analysis. In order to describe the characteristics of the sample, means and standard deviations were used for continuous variables and frequencies and percentages for categorical variables. For the comparison of baseline characteristics between the institutionalized group (IG) and the group of social-health care personnel (GPSS), Student’s *t*-test was used for continuous variables and Pearson’s Chi-squared test (χ2) for categorical variables. To explore the association between vaccine immunity production concerning age and BMI of the participants, Spearman’s nonparametric correlation test was used after checking using the Shapiro–Wilk test for the data that did not fit a normal distribution. Finally, Kruskal–Wallis test was used to evaluate possible differences between the production of immunity concerning the blood group and the number of diseases of the participants. For all analyses, a two-tailed *p*-value < 0.05 was considered significant. On the other hand, the *t*-test was used to determine the existence of significant differences in adverse reactions between the first and second doses. A *p*-value < 0.05 was considered significant.

### 2.8. Ethical Considerations

The study was approved by the Ethics Committee of the Valladolid East Health Area of University Clinical Hospital of Valladolid (Valladolid, Spain) with PI No. 21-2413. All the participants were given informed consent before taking part.

## 3. Results

### 3.1. Sample and Lifestyle Characteristics

Among the 112 eligible participants invited to participate in the study, four (3.6%) healthcare workers were excluded (three refused vaccination and one pregnant), and two (1.8%) institutionalized patients were excluded (only received the first dose of the vaccine). Therefore, the remaining 106 patients fulfilled the inclusion/exclusion criteria and comprised the final study sample. The sample consisted of 37 men (34.9%) and 69 (65.1%) women, and 31 (93.9%) women were in the healthcare workers group. The mean age of institutionalized patients was 84.3 ± 7.6 years, and of healthcare workers, 48.8 ± 12.8; most participants were born in Spain (98.1%). Regarding lifestyle-related characteristics, the mean BMI of 27.2 ± 5.2 kg/m^2^ evidenced that the sample was overweight according to SEEDO criteria [24]. A total of 58.5% were non-smokers; this percentage was higher in institutionalized patients (71.2%). However, regarding healthcare workers, 39.4% were smokers. The proportion of adherent patients to the Mediterranean diet according to the Trichopoulou et al. [25] questionnaire was 10.1 ± 1.8. The total sample reported 79.4 ± 16.1 of self-perceived well-being, as assessed by the Visual Analogue Scale (VAS) adapted by Gould et al. [26]. There were significant differences (*p* = 0.019) between institutionalized patients (76.9 ± 15.7) and healthcare workers (84.8 ± 15.8) (Table 1).

### 3.2. Physical Fitness Condition

For institutionalized patients, the handgrip strength was at sarcopenic stages; according to the European Working Group on Sarcopenia in Older People (EWGSOP) [29], the handgrip strength was 16.10 ± 11.70 kg/cm^2^ and 13.0 ± 8.90 kg/cm^2^ in the dominant hand and the non-dominant hand respectively. Furthermore, 32.9% of the institutionalized patients were at risk assessment for falls according to the “*Get Up and Go Test*” [28], while 6.9% were unable to perform the test due to their disability. In healthcare workers, handgrip strength was within the appropriate values [29] for their age range (30.0 ± 13.7), and none were at risk of falling (Table 1).

### 3.3. Clinical Description

None of the 106 study participants were infected by SARS-CoV-2 before or during the study. Only one institutionalized patient used oxygen therapy, and 24.5% of the participants suffered from drug allergies. Arterial hypertension (44.3%) were the most chronic conditions among study participants, followed by obesity (33.0%), cardiovascular (33.0%), cancer (26.4%), insulin-dependent diabetes mellitus (22.6%), and respiratory disease (14.2%). The percentage of illnesses such as cancer (39.4%) and respiratory diseases (18.2%) was higher in healthcare workers. The usual pharmacological treatments used by the study participants were mostly anxiolytics/sedatives (50.9%), followed by antihypertensives (48.1%), cardiovascular drugs (47.2%), anticoagulants (23.6%), antidiabetics (14.2%), lipid-lowering agents (10.4%) and immunosuppressants (0.9%). None of the healthcare workers took anticoagulant drugs, immunosuppressants, lipid-lowering agents, or antidiabetics (Table 1).

### 3.4. Vaccination Process

A total of three lots of BNT162b2 vaccines (Conmirnaty^®^) belonging to the Pfizer/BioNTech company were administered to the study participants. The first dose of the vaccine was administered on 13 January 2021, lot EM477 being administered to 98.1% of the participants, while only 1.9% were administered a different lot (EK9788). During the second dose completing the vaccination schedule 21 days after the first dose (3 February 2021), 98.1% of participants received the EK9788 lot dose, while only 1.9% were administered a different lot (ES3964). All volunteers (*n* = 106) were vaccinated in the non-dominant arm.

### 3.5. BNT162b2 Vaccine-Related Adverse Reactions

#### 3.5.1. Local Reactions

Among the participants, pain at the injection site of the first (44.3%) and second dose (21.6%) of the vaccine was the most frequent local reaction, followed by redness (first 18.8%/second 17.9%) and swelling (first 18.8%/second 13.3%). Four participants in the study declared moderate pain after the first dose, and one patient reported serious pain after the second dose (Table 2 and Table 3). Significant differences (*p*-value < 0.05) were observed in pain at the injection site between the first and second dose of the BNT162b2 vaccine (Supplementary Material Table S1).

#### 3.5.2. Systemic Reactions

As shown in Table 2 and Table 3, the systemic adverse reactions for both doses, most reported by study participants, were headache (first 28.2%/second 20.7%), muscle aches or pains (first 28.2%/second 17.9%), and fatigue or tiredness (first 22.5%/second 15.0%). None of the participants reported severe systemic adverse reactions, but for the first dose 3 patients reported serious reactions for fatigue (*n* = 1), fever (*n* = 1) and headache (*n* = 1) and 8 patients after the second dose, reporting fatigue (*n* = 2), muscle aches (*n* = 1), joint pains (*n* = 3) and headache (*n* = 2). No significant differences (*p*-value > 0.05) were observed in systemic adverse reactions between the first and second dose of the BNT162b2 vaccine (Supplementary Material Table S1).

### 3.6. Antibody Level

**Figure 3:**
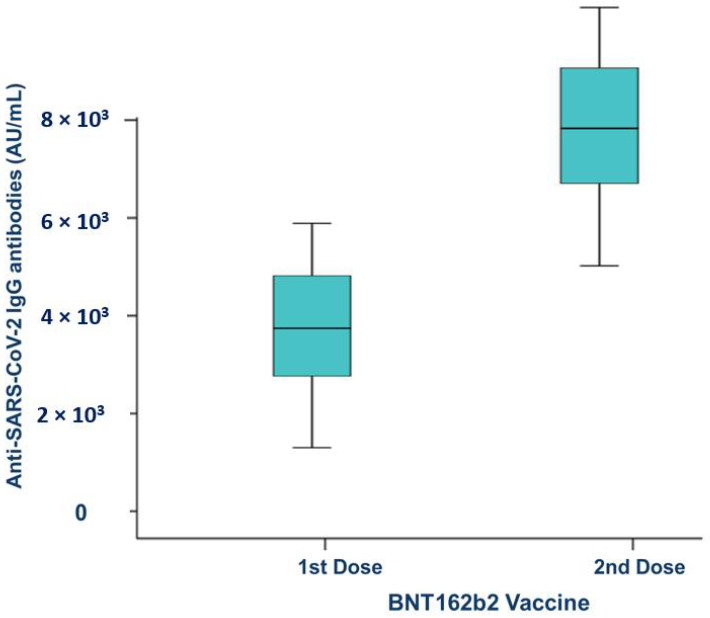
Antibody level generated by the BNT162b2 vaccine.

Both vaccine doses elicited anti-SARS-CoV-2 IgG antibodies after the first dose (3799.0 ± 2503.0 AU/mL; median 4083 AU/mL) and second dose (8212.0 ± 4731.0 AU/mL; median 8345 AU/mL), which were assessed 20 days after each dose of BNT162b2 vaccine (Figure 3).

### 3.7. Associations between Antibody Titer and Participant Characteristics

#### 3.7.1. Age

**Figure 4:**
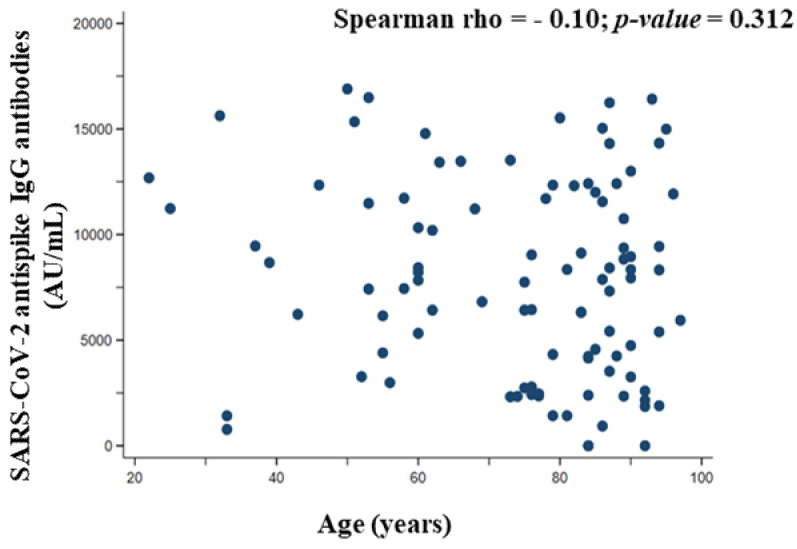
Correlation between total SARS-CoV-2 anti-spike IgG antibody titer (AU/mL) quantified 20 days after administration of the second dose of vaccine and age of participants.

Figure 4 shows the correlation between the total SARS-CoV-2 anti-spike IgG antibodies titer (quantified 20 days after the second dose of vaccine) and the age of the study participants. The correlation coefficient between titer and age (Spearman rho = −0.10, *p*-value = 0.312) shows a negative association, but this relationship was of very weak intensity and not statistically significant.

#### 3.7.2. Body Mass Index

**Figure 5:**
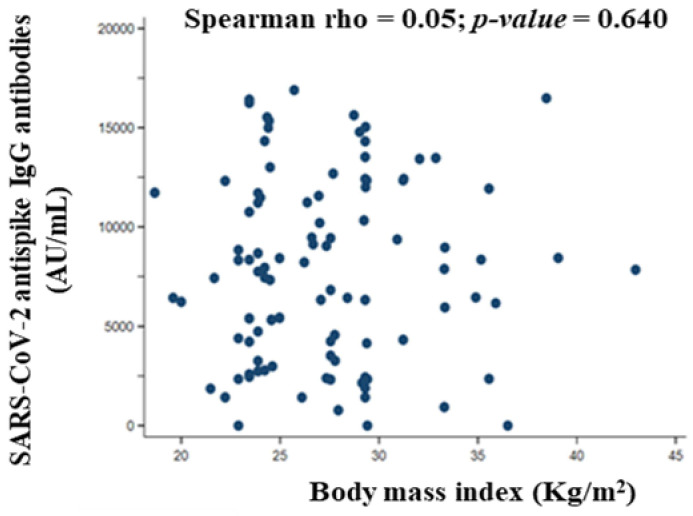
Correlation between total SARS-CoV-2 anti-spike IgG antibody titer (AU/mL) quantified 20 days after administration of the second dose of vaccine and body mass index of participants.

The correlation coefficient between total SARS-CoV-2 anti-spike IgG antibodies titer and BMI (Spearman rho = 0.05, *p*-value = 0.640) showed a positive association, but this relationship was extremely weak and not statistically significant (Figure 5).

#### 3.7.3. Number of Drugs and Specific Treatments

The association between total SARS-CoV-2 anti-spike IgG antibodies titer and the number of drugs was evaluated, and an extremely weak negative correlation without statistical significance was observed (Spearman rho = −0.07, *p*-value = 0.490). Furthermore, the comparative analysis through the nonparametric test of two independent samples (Mann–Whitney U) showed that there were no significant differences between participants with antihypertensive (*p*-value > 0.05), immunosuppressant (*p*-value > 0.05), antidiabetic (*p*-value > 0.05), and lipid-lowering (*p*-value > 0.05) treatment and those participants without the prescription of these treatments (data not shown graphically).

#### 3.7.4. Blood Group

**Figure 6:**
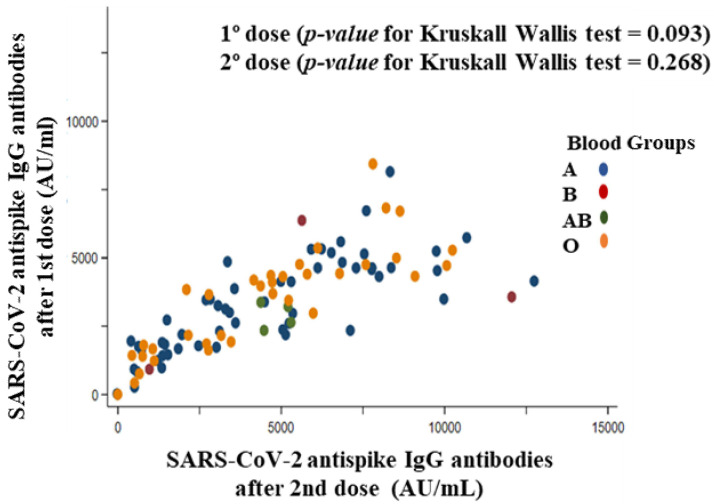
Correlation between total SARS-CoV-2 anti-spike IgG antibody titer (AU/mL) after the first dose and IgG antibody titer (AU/mL) after the second dose stratified by blood group of the participants.

Figure 6 represents the correlation between SARS-CoV-2 anti-spike IgG antibodies titer after the first dose and total SARS-CoV-2 anti-spike IgG antibodies titer after the second dose (total antibodies—antibodies generated in the first dose) stratified by blood group of the participants. The volunteers (*n* = 106) were represented by a colored dot according to the blood group (A = blue; B = red; AB = green; O = orange). The comparison between the four blood groups was not significant for both antibody titer after the first dose (*p*-value for Kruskal–Wallis test = 0.093) and antibody titer after the second dose (*p*-value for Kruskal–Wallis test = 0.268). The four blood groups (A, B, AB, O) of study participants did not follow a defined pattern, but there was a high degree of dispersion (Figure 6).

#### 3.7.5. Chronic Conditions

**Figure 7:**
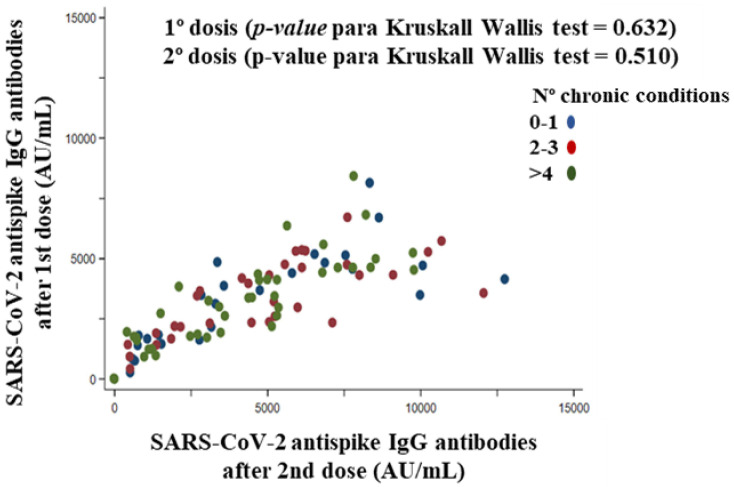
Correlation between SARS-CoV-2 anti-spike IgG antibody titer (AU/mL) after the first dose and IgG antibody titer (AU/mL) after the second dose stratified by the number of chronic conditions of the participants.

The comparison between the three chronic conditions groups (0–1 = blue; 2–3 = red; ≥4 = green), for the volunteers (*n* = 106), was non-significant for both anti-SARS-CoV-2 antibodies titer after the first dose (*p*-value for Kruskal–Wallis test = 0.632) and anti-SARS-CoV-2 antibodies titer after the second dose (*p*-value for Kruskal–Wallis test = 0.510). The three chronic condition groups did not follow a defined pattern, but rather there was a high degree of dispersion (Figure 7).

### 3.8. Association between the First and Second Doses of BNT162b2 Vaccine Concerning SARS-CoV-2 Anti Spike IgG Antibodies Titer in Participants

**Figure 8:**
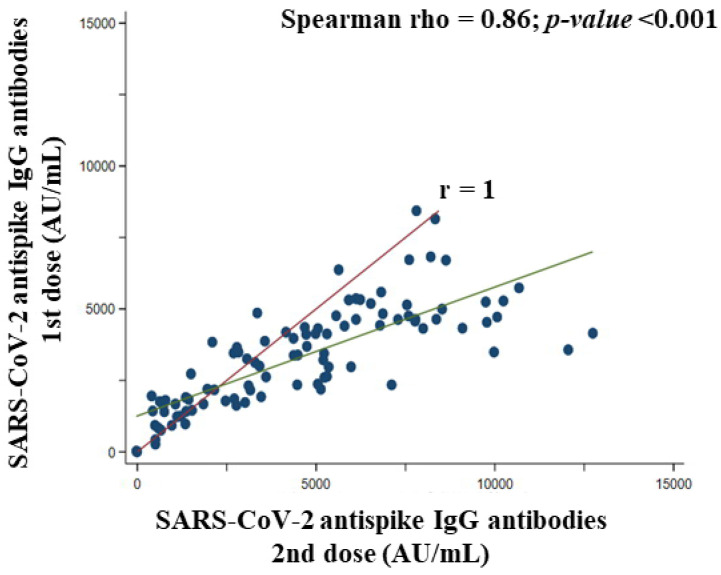
Correlation between SARS-CoV-2 anti-spike IgG antibody titer (AU/mL) after the first dose and after the second dose of BNT162b2 vaccine.

The green line in Figure 7 corresponds to the correlation fit line between SARS-CoV-2 anti-spike IgG antibodies titer after the first dose and SARS-CoV-2 anti-spike IgG antibodies titer for the second dose (Spearman rho = 0.86, *p*-value < 0.001) which showed a very strong and positive association. Thus, the higher the SARS-CoV-2 anti-spike IgG antibodies titer created in the first dose, the higher the titer in the second dose of the *BNT162b2 vaccine*. Furthermore, it can be seen how most of the volunteers (points below the red line which is the fit line of a perfect correlation [r = 1]) developed a higher SARS-CoV-2 anti spike IgG antibodies titer after the second dose (total antibodies—antibodies generated in the first dose) than at the first dose (Figure 8).

### 3.9. Association between SARS-CoV-2 Anti Spike IgG Antibodies Titer of the First Dose and Total SARS-CoV-2 Anti Spike IgG Antibodies Titer of the Participants

**Figure 9:**
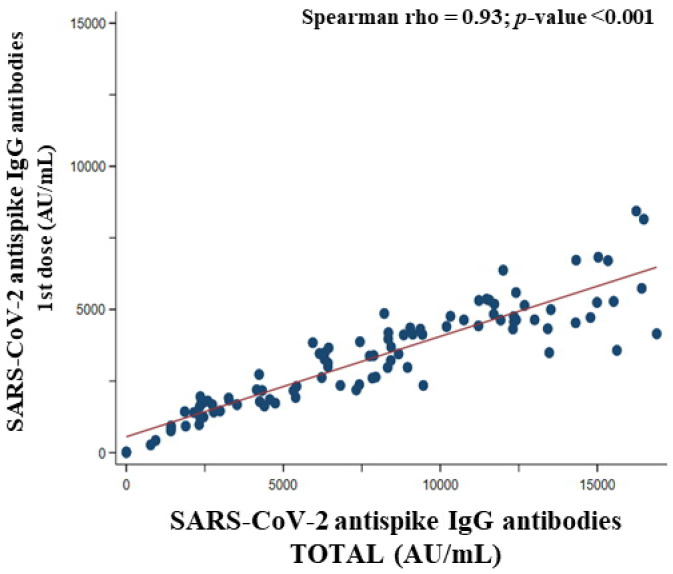
Correlation between SARS-CoV-2 anti-spike IgG antibody titer (AU/mL) after the first dose of BNT162b2 vaccine and total SARS-CoV-2 anti-spike IgG antibody titer.

Figure 9 represents the correlation between SARS-CoV-2 anti-spike IgG antibodies titer after the first dose and total SARS-CoV-2 anti-spike IgG antibodies titer. The red-colored line corresponds to the fit line of the correlation between titers (Spearman rho = 0.93, *p*-value < 0.001), demonstrating a positive and very strong association. In consequence, the higher the titer of SARS-CoV-2 anti spike IgG antibodies titer created in the first dose, the higher the total SARS-CoV-2 anti spike IgG antibodies titer.

## 4. Discussion

Our findings, obtained with an independent study, suggest that two doses of Pfizer BioNTech (BNT162b2) COVID-19 vaccine (Conmirnaty^®^) induce an immune response with the production of anti-SARS-CoV-2 IgG antibodies in 100% of participants, regardless of interindividual characteristics (age, BMI, blood group, number of drugs, number of chronic diseases), and none of them having a confirmed history of SARS-CoV-2 infection or a positive nasopharyngeal test during the study. SARS-CoV-2 anti-spike IgG antibodies were intensely elevated after the second administration of the BNT162b2 vaccine. We report that the higher the titer of anti-SARS-CoV-2 IgG antibodies generated after the first dose of vaccine, the higher the titer of anti-SARS-CoV-2 IgG antibodies generated by the second dose of vaccine and the total antibody titer of anti-SARS-CoV-2 IgG antibodies. As well, no severe adverse effects were reported among participants, although mild or moderate adverse effects (local or systemic) were reported after both doses of the BNT162b2 vaccine, being more frequent after the first dose of the vaccine.

Immunosenescence is age-related and causes dysregulation of the immune system, leading to poor responses to vaccination. Furthermore, qualitative differences in memory B cells and differentiation into plasma cells have been observed in older adult patients, leading to impaired protection after immunization [30]. In this sense, recent studies [30,31,32,33,34] reported that increasing age reduces the effectiveness of an immunization against COVID-19. Likewise, Walsh et al. [35] showed a significantly lower anti-SARS-CoV-2 IgG antibodies titer in adults aged 65 to 85 years than in adults aged 18 to 55 years at 21 days after administration of the first dose of the vaccine. However, this difference in anti-SARS-CoV-2 IgG antibodies titers, between 18–55 vs. 65–85 years, was lower after 7 days of the second dose of Vaxzevria^®^ (AstraZeneca, Cambridge, UK; ChAdOx1-S [recombinant]), Spikevax^®^ (Moderna Cambridge, Massachusetts, USA; INN-COVID-19 mRNA [nucleoside modified]), Janssen^®^ (Janssen, Beerse, Belgium; Ad26.COV2.S [recombinant]) or Conmirnaty^®^ vaccines. Furthermore, Pellini et al. [36] and Mitsunaga et al. [37] described that the total anti-SARS-CoV-2 IgG antibody titer was significantly reduced in participants >60 years after the complete vaccine regimen. Furthermore, Muller et al. [38] showed differences between the total antibody responses generated after the first and/or second BNT162b2 vaccine, with lower frequencies of neutralizing antibodies in elderly patients (>80 years). In contrast to these earlier studies, our results (Figure 4) showed that there was no unison variation between the total SARS-CoV-2 anti-spike IgG antibodies and the age of the participants. These findings might indicate that there was no relationship between the total IgG titer and the age of the participants. A balanced immune response of the study participants could be a plausible explanation. The volunteers have shown a high adherence to the Mediterranean diet. The Mediterranean diet contains nutrients such as polyphenols, phytochemicals, monounsaturated and polyunsaturated fatty acids, zinc, calcium, vitamin C, vitamin E, and vitamin D have shown immunostimulant effects [39]. Specifically, vitamin D plays an essential role in immune systems [40], and vitamin D deficiency is quite common among COVID-19 patients [41], so vitamin D administration could reduce the risk of incidence and death from COVID-19 at appropriate doses [42]. Vitamin D supplementation improves influenza vaccine response and immune function in elderly persons [43]. In addition, Kashi et al. [44] reported a positive association between plasma vitamin D levels and hepatitis B antibody titer following a complete vaccination. Similarly, Vitamin D has been used as an adjuvant in Bacillus Calmette-Guérin (tuberculosis) vaccination with moderate benefits on induced immunity [45]. In this sense, a higher adherence to the Mediterranean diet could be key to improving the immune system and vaccine response [46] because vitamin D could act by modulating vaccine-induced cytokine and inflammatory messenger responses [40].

The efficacy of COVID-19 vaccines in overweight/obese persons is problematic, given that 39% of adults (≥18 years) are overweight, and 13% are obese [47]. Obesity and overweight can weaken immune responses and reduce antibody production after vaccination, such as over influenza, hepatitis B, rabies, and tetanus vaccines [36]. However, the results for the BNT162b2 vaccine are controversial. Mitsunaga et al. [37] reported that obese participants (BMI ≥ 30) had a lower antibody titer than compared to those of normal weight. However, Pellini et al. [36] did not confirm these results, as in our study with overweight grade II (pre-obese) participants by SEEDO criteria [24]. Minimal levels of inflammation and reasonably elevated levels of inflammatory cytokines induced by adipose tissue in overweight individuals may weaken immune responses [48]. The anti-inflammatory action of nutrients in the Mediterranean diet, more specifically, monounsaturated and polyunsaturated (ω-3) fatty acids, could be responsible for the beneficial effects on immune system enhancement [46]. In this sense, ω-3, as adjuvants, have the potential to reduce the morbidity and mortality of SARS-CoV-2 infection [49].

Immune declines associated with chronic diseases are of particular concern in the elders and may be associated with differences in immune responses to vaccines [50]. Inadequate response to influenza or pneumococcal vaccines was observed in groups of patients with a high prevalence of chronic diseases [48,50]. Mitsunaga et al. [37] observed that the proportion of chronic lung diseases, hypertension, diabetes, dyslipidemia, autoimmune diseases, and cancer was significantly higher in the group of participants with lower antibody titer after two doses of the BNT162b2 vaccine. In addition, hypertension and/or diabetes (glycosylated hemoglobin [HbA1c] > 6.5%) were significant suppressors of antibody responses. Furthermore, a high percentage of patients with hematologic malignancies who have received two doses of the BNT162b2 vaccine were seronegative, and seropositive patients have moderately low titers compared to healthy subjects [51]. Contrary to previous investigations, we demonstrated that the number of chronic conditions did not influence antibody titer after the first dose and second dose of the BNT162b2 vaccine.

Regarding the drugs used for the treatment of the chronic conditions afflicted on the study participants, we did not observe significant differences in the humoral response to Conmirnaty^®^ vaccine between participants with antihypertensive, immunosuppressive, antidiabetic, and lipid-lowering treatments and those participants without a prescription for previous treatments. Thus, all patients with pharmacological treatment produced SARS-CoV-2 anti-spike IgG antibodies. Most drugs taken by patients do not affect the immune system, but some suppress the action of the immune system against infections and develop a poor serological response to vaccination. Recently, it has been described that the humoral response to mRNA vaccines (Comirnaty^®^ or Spikevax^®^) was severely impaired in patients on B-cell targeted therapies (either Rituximab or Ibrutinib) [52]. Intravenous infusion of convalescent plasma therapy has been proposed as an alternative for immunization of these patients. Convalescent plasma therapy was used successfully in other viral outbreaks in the 20th century [53].

Literature data suggest that there is a significant association between blood group A and a worse prognosis of COVID-19, while group 0 patients have a significantly lower risk of infection. The S protein of SARS-CoV-2 has a similar structure to that of the ABO blood groups, and this means that when SARS-CoV-2 infects a person of blood group 0, the immune system reacts by using the antibodies (anti-A and anti-B) in the blood to attack SARS-CoV-2, which would hinder its spread in the host cells [54]. Furthermore, blood group antigens could act as pathogen receptors and take part in immune cell interactions [55]. In addition, Rh-negative patients had a lower risk of viral infection, severe disease, and mortality after SARS-CoV-2 infection [56,57]. In the context of the above, blood group-related differences in the humoral response to the SARS-CoV-2 vaccine might be expected. However, no differences were observed between the blood groups for antibody titer after the first dose and second dose of the BNT162b2 vaccine. Therefore, ABO/Rh blood group system does not support a predictive model of immunogenicity of the BNT162b2 vaccine, just as ABO/Rh screening should not be used as a triage mechanism in COVID-19 [58].

In our study, the first dose stimulated SARS-CoV-2 anti-spike IgG antibodies generation, but it was of a lower magnitude than the second dose of BNT162b2. Thus, second homologous immunization would improve the humoral immune response with an adequate safety profile since the adverse reactions after the administration of the first dose of the BNT162b2 vaccine were greater than after the second dose. Thompson et al. [59] demonstrated that administration of the one-dose SARS-CoV-2 mRNA vaccine achieved 80% immunization, but it was after the two-dose that 90% of participants reached it. Conmirnaty^®^ or Spikevax^®^ are highly effective in preventing SARS-CoV-2 infection, attenuated viral RNA load, risk of febrile symptoms, and duration of illness among those who had progression of infection despite vaccination [59]. We reported no SARS-CoV-2 infection in the study volunteers from baseline to 21 days after administration of the second dose of the BNT162b2 vaccine. In addition, we have observed that the greater the IgG response after the first dose, the greater the IgG response after the second dose, and the greater the total immunogenicity. Therefore, the humoral immune response would be substantially amplified, which is key for Comirnaty^®^ to be effective since, in respiratory infections, a higher titer of antibodies is necessary for adequate immune protection [60] and particularly important for the revaluation of temporal distancing of the interval between doses in some vaccination strategies of the health authorities [61]. Liu et al. [62] reported immune sera obtained after two doses of BNT162b2 (2–4 weeks) have elevated antibody titers against different variants B.1.617.1, B.1.618 (first identified in India), and B.1.525, being especially elevated against the Delta variant B.1.617.2 lineage. Furthermore, Wang et al. [63] have described that 8 weeks after the second dose of Conmirnaty^®^ or Spikevax^®^, volunteers showed high levels of SARS-CoV-2 anti spike IgG and IgM and RBD binding titer. In this study, Wang et al. [63] plasma neutralizing activity and relative numbers of RBD-specific memory B lymphocytes of vaccinated volunteers were equivalent to those of individuals who had recovered from likely SARS-CoV-2 infection. Two studies in elderly adults [64] and in aged mice [65] showed that granzyme B stimulation following influenza and SARS-CoV-2 (ChAdOx1 nCoV-19) booster vaccination, respectively, would provide an enhanced immune response by reactivating granzyme B CD8 T cell activity. In this way, our results indicate that Conmirnaty^®^, a licensed COVID-19 mRNA vaccine, was effective in preventing SARS-CoV-2 infection with the dual vaccination regimen in institutionalized patients and healthcare workers, which are two of the social groups most in need of an effective vaccine to prevent COVID-19.

Participants have reported local and systemic adverse reactions after mRNA-based vaccination [19,65,66,67]. In our study, the incidence of adverse reactions was higher after the first dose than after the second dose. However, other studies [65,66] reported a higher incidence of adverse reactions observed after the second dose of mRNA-based vaccine. In contrast with Kitagawa et al. [68] and Saita et al. [69], we showed a significant difference in injection site pain between the first and second dose. The most frequent systemic adverse reaction reported in the literature has been fatigue [19,65,67,70], but in our study, it was headache and joint pain. For healthcare workers who received mRNA-based COVID-19 vaccines [71,72], the reported side effects were like those described in our study. Therefore, pain at the injection site was the most common local side effect; in addition, others such as headache/fatigue, muscle pain, chills, and joint pain were reported as the most common side effects [71,72], as is the case in our study. However, Klugar et al. [72] reported at least one oral side effect, including mucosal lesions, oral paresthesia, and taste disturbance, that was not reported in our study. No severe adverse reactions were reported, but three participants after the first dose and nine after the second dose reported serious grade reactions. This could be because the volunteers lacked a record of previous COVID-19 infection, which would attenuate the incidence and intensity of side effects of vaccination [73].

This study has several limitations. First, given the nature of the self-reporting survey, the frequency of reported adverse reactions, adherence to the Mediterranean diet, and self-perception of health status may have been over-or underestimated. However, the reported adverse reactions were medically verified. Second, the sample size was small and included predominantly institutionalized patients with a mean age of approximately 85, with healthcare workers aged above 50 years old being less represented. Third, the demonstration of final efficacy and safety of the dual dose of BNT162b2 in COVID-19 for no infected subjects is limited in this study to a 21-day follow-up after the second vaccination dose. Fourth, we only analyzed the quantitative serological response to the dual dose of the Pfizer-BioNTech vaccine without considering the cellular response.

In conclusion, our findings suggest that the BNT162b2 vaccine has demonstrated adequate SARS-CoV-2 anti-spike IgG titer independent of age, BMI, blood group, number of chronic diseases, and pharmacological treatment. The second dose induced a higher number of SARS-CoV-2 anti-spike IgG antibodies than the first dose and a booster immunity effect. Adverse reactions, local and systemic, were mostly mild or moderate without severe symptoms and most frequently after the first dose of the BNT162b2 vaccine. Further studies will be needed to assess the long-term immunogenicity and safety of the two doses of mRNA vaccines and even in previously infected persons, to better implement vaccination plans.

## Figures and Tables

**Table 1:** Antibody Levels, Sociodemographic and Lifestyle, Physical Fitness, and Clinical Characteristics-Related with study participants.

Characteristics	Full Cohort (*n* = 106)	Institutionalized Patients (*n* = 73)	Healthcare Workers (*n* = 33)	*p*-Value
**Sociodemographic and Lifestyle**				
Gender, *n* (%)				<0.001
Male	37 (34.9)	35 (47.9)	2 (6.1)	
Female	69 (65.1)	38 (52.1)	31 (93.9)	
Age (years), mean (SD)	73.3 (19.1)	84.3 (7.6)	48.8 (12.8)	<0.001
Nationality, *n* (%)				0.034
Spanish	104 (98.1)	73 (100.0)	31 (93.9)	
Other	2 (1.9)	0	2 (6.1)	
^1^ Body mass index (BMI), mean (SD)	27.2 (5.2)	27.3 (4.0)	26.9 (7.1)	0.751
Smoker, *n* (%)	19 (17.9)	6 (8.2)	13 (39.4)	<0.001
Non-Smoker	25 (23.6)	15 (20.5)	10 (30.3)	
Never Smoker	62 (58.5)	52 (71.2)	10 (30.3)	
^2^ Trichopoulou’s MedDiet score, mean (SD)	10.2 (1.8)	10.1 (1.5)	10.3 (2.4)	0.610
^3^ Self-perceived health status g (%), mean (SD)	79.4 (16.1)	76.9 (15.7)	84.8 (15.8)	0.019
**Physical Fitness**				
^4^ Manual pressure dynamometry (kg/cm^2^), mean (SD)				
Dominant hand	20.4 (13.9)	16.1 (11.7)	30 (13.7)	<0.001
Non-dominant hand	17.6 (12.3)	13 (8.9)	28 (12.5)	<0.001
^5^ Get-Up-And-Go Test (seconds), *n* (%),				
Yes (>30 seg)	24 (22.6)	24 (32.9)	0	
No	77 (72.6)	44 (60.3)	33 (100.0)	---
Disabled	5 (4.7)	5 (6.9)	0	
**Clinics**				
Known allergies, *n* (%)				0.963
Yes	26 (24.5)	18 (24.7)	8 (24.2)	
No	80 (75.5)	55 (75.3)	25 (75.8)	
^6^ Previously passed COVID-19 infection, *n* (%)				0.231
Yes	0	0	0	
No	106 (100)	73 (100)	33 (100)	
Chronic conditions, *n* (%)				
Arterial hypertension	47 (44.3)	38 (52.1)	9 (27.3)	0.017
Obesity	35 (33.0)	28 (38.4)	7 (21.2)	0.082
Insulin-dependent diabetes mellitus	24 (22.6)	20 (27.4)	4 (12.1)	0.082
^7^ Respiratory	15 (14.2)	9 (12.3)	6 (18.2)	0.423
Cancer	28 (26.4)	15 (20.5)	13 (39.4)	0.042
^8^ Cardiovascular	35 (33.0)	30 (41.1)	5 (15.2)	0.009
Usual treatment, *n* (%)				
Antihypertensives	51 (48.1)	39 (53.4)	12 (36.4)	0.104
Anticoagulants	25 (23.6)	25 (34.2)	0	<0.001
Immunosuppressants	1 (0.9)	1 (1.4)	0	0.499
Anxiolytics/Sedatives	54 (50.9)	52 (71.2)	2 (6.1)	<0.001
Lipid lowering agents	11 (10.4)	11 (15.1)	0	0.018
Antidiabetics	15 (14.2)	15 (20.5)	0	0.005
Cardiovascular	50 (47.2)	47 (64.4)	3 (9.1)	<0.001
Use of oxygen therapy, *n* (%)				0.728
Currently	1 (0.9)	1 (1.4)	0	
Previous/Occasional	5 (4.7)	3 (4.1)	2 (6.1)	
Never	100 (94.3)	69 (94.5)	31 (93.9)	
Vital signs, mean (SD)				
Blood pressure				
SBP (mmHg)	126 (15.0)	127 (15.0)	123 (15.0)	
DBT (mmHg)	71.3 (13.4)	70.1 (14.7)	73.9 (9.4)	
Heart rate (bpm)	75.1 (11.7)	74.3 (12.3)	76.9 (10.1)	
Temperature (°C)	35.8 (0.5)	35.9 (0.4)	35.7 (0.5)	
Oxygen saturation (%)	96.9 (1.7)	96.4 (1.6)	98 (1.3)	

Abbreviations: COVID-19, coronavirus 2019; SD, standard deviation; kg, kilograms; mmHg, millimeters of mercury; bpm, beats per minute; DBT, diastolic blood pressure; SBP, systolic blood pressure; °C, degrees Celsius. Values are expressed as mean (SD) for quantitative variables and as frequency (percentage) for categorical variables. ^1^ Results obtained according to Spanish Obesity Society (SEEDO) criteria [24]; ^2^ Score proposed by Trichopoulou et al. [25]; ^3^ Assessed by Visual Analogue Scale (VAS) adapted from Gould et al. [26]; ^4^ Dynamometer Measurements described by Bohannon [27]; ^5^ Fall risk measurement assessment using the “Get up and go” test proposed by Gálvez Cano et al. [28]. Those classified as disabled were unable to perform the test because they were bedridden or wheelchair users; ^6^ Laboratory confirmed positive case by RT-PCR as explained by Fernandez et al. [23]; ^7^ Including respiratory failure, chronic obstructive pulmonary disease, asthma, and cystic fibrosis; ^8^ Including coronary heart disease, heart failure, venous and/or arterial insufficiency and stroke.

**Table 2:** Symptoms experienced after the first vaccination with BNT162b2 (Pfizer/BioNTech) vaccine (Conmirnaty^®^).

Type	Symptom	Presence of Symptoms	Symptoms Score			
		Participants (*n* = 106)	1	2	3	4
		*n* (%)	*n* (%)	*n* (%)	*n* (%)	*n* (%)
Local reactions	Injection site Pain	47 (44.3)	43 (90.9)	4 (9.1)	-	-
	Injection site Redness	20 (18.8)	20 (100.0)	-	-	-
	Injection site Swelling	20 (18.8)	18 (90.4)	2 (9.6)	-	-
Systemic reactions	Chills or shivering	18 (17.0)	16 (89.4)	2 (10.3)	-	-
	Fatigue or tiredness	24 (22.5)	18 (75.1)	5 (20.8)	1 (4.1)	-
	Muscle aches or pains	30 (28.2)	23 (76.9)	6 (19.8)	1 (3.3)	-
	Headache	30 (28.2)	22 (73.5)	8 (26.5)	-	-
	Joint pains	15 (14.0)	14 (93.6)	1 (6.4)	-	-
	Vomiting or Nauseous	9 (8.4)	8 (93.7)	1 (6.3)	-	-
	Diarrhea	11 (10.0)	7 (64.1)	4 (35.9)	-	-
	Fever (≥38.0 °C)	4 (3.2)	2 (40.6)	1 (29.7)	1 (29.7)	-

Symptoms Score 1: Mild; 2: Moderate; 3: Serious; 4: Severe.

**Table 3:** Symptoms experienced after the second vaccination with BNT162b2 (Pfizer/ BioNTech) vaccine (Conmirnaty^®^).

Type	Symptom	Presence of Symptoms	Symptoms Score			
		Participants (*n* = 106)	1	2	3	4
		*n* (%)	*n* (%)	*n* (%)	*n* (%)	*n* (%)
Local reactions	Injection site Pain	23 (21.6)	20 (87.1)	2 (8.3)	1 (4.6)	-
	Injection site Redness	19 (17.9)	18 (94.9)	1 (5.1)	-	-
	Injection site Swelling	14 (13.3)	14 (100)	-	-	-
Systemic reactions	Chills or shivering	14 (13.3)	11 (78.9)	3 (21.1)	-	-
	Fatigue or tiredness	16 (15.0)	12 (75.4)	2 (12.3)	2 (12.3)	-
	Muscle aches or pains	19 (17.9)	11 (58.1)	7 (36.9)	1 (5.0)	-
	Headache	22 (20.7)	14 (63.7)	6 (27.1)	2 (9.2)	-
	Joint pains	11 (10.3)	5 (45.6)	3 (27.2)	3 (27.2)	-
	Vomiting or Nauseous	5 (4.6)	4 (80.4)	1 (19.6)	-	-
	Diarrhea	7 (6.5)	5 (72.3)	2 (27.7)	-	-
	Fever (≥38.0 °C)	1 (0.9)	1 (100.0)	-	-	-

Symptoms Score 1: Mild; 2: Moderate; 3: Serious; 4: Severe.

## Data Availability

Not applicable.

## Supplementary Materials

The following supporting information can be downloaded at: https://www.mdpi.com/article/10.3390/vaccines10081170/s1, Table S1. Comparison of adverse reactions after propensity score matching by the number of doses of BNT162b2 (Pfizer/BioNTech) vaccine (Conmirnaty^®^) vaccine administered.
