# Supplementary material for: Effectiveness of Comirnaty® Vaccine and Correlates of Immunogenicity and Adverse Reactions: A Single-Center Prospective Case Series Study
Source: Vaccines (Basel). 2022 Jul 22;10(8):1170. doi: 10.3390/vaccines10081170 (PMC9330441; doi:10.3390/vaccines10081170)
Supplement: Supplementary file 1 [file vaccines-10-01170-s001.zip › vaccines-1784836-supplementary.pdf]

Supplementary Table S1. Comparison of adverse reactions after propensity score matching by the number of doses of BNT162b2 (Pfizer/BioNTech) vaccine (Conmirnaty®) vaccine administered.

**Type**	**Symptom**	**Presence of Symptoms One-Dose**	**Presence of Symptoms Two-Dose**	***p* Value**
		**Participants (n = 106)**		
		**n (%)**		
**Local Reactions**	Injection site Pain	47 (44.3)	23 (21.6)	<0.05
	Injection site Redness	20 (18.8)	19 (17.9)	>0.05
	Injection site Swelling	20 (18.8)	14 (13.3)	>0.05
**Systemic Reactions**	Chills or shivering	18 (17.0)	14 (13.3)	>0.05
	Fatigue or tiredness	24 (22.5)	16 (15.0)	>0.05
	Muscle aches or pains	30 (28.2)	19 (17.9)	>0.05
	Headache	30 (28.2)	22 (20.7)	>0.05
	Joint pains	15 (14.0)	11 (10.3)	>0.05
	Vomiting or Nauseous	9 (8.4)	5 (4.6)	>0.05
	Diarrhea	11 (10.0)	7 (6.5)	>0.05
	Fever (≥38.0 °C)	4 (3.2)	1 (0.9)	>0.05
